# Role of functional single nucleotide polymorphisms of MMP1, MMP2, and MMP9 in open angle glaucomas

**Published:** 2010-08-28

**Authors:** Georg Mossböck, Martin Weger, Christoph Faschinger, Christine Zimmermann, Otto Schmut, Wilfried Renner, Yosuf El-Shabrawi

**Affiliations:** 1 Department of Ophthalmology, Medical University of Graz, Graz, Austria; 2 Clinical Institute of Medical and Chemical Laboratory Diagnostics, Medical University of Graz, Graz, Austria

## Abstract

**Purpose:**

Matrix metalloproteinases (MMPs) play an essential role in the turnover of the extracellular matrix and cellular behavior. MMP1, MMP2, and MMP9 have previously been implicated in the pathogenesis of primary open angle glaucoma (POAG) and open angle glaucoma secondary to exfoliation syndrome (XFG), respectively. Functional gene polymorphisms of these MMPs such as *MMP1* −1607 1G/2G (rs1799750), *MMP2* −1306 C/T (rs243865), *MMP2* −1575 G/A (rs243866), and *MMP9* Q279R (rs17576) are thus plausible candidates as risk factors for open angle glaucomas. The purpose of the present study was to investigate hypothesized associations between these polymorphisms and the presence of POAG and XFG in a Caucasian population.

**Methods:**

The present case-control study included 322 patients with POAG, 202 patients with XFG, and 248 control subjects. Genotyping of polymorphisms was done using polymerase chain reaction.

**Results:**

No significant differences in either genotype distributions or allelic frequencies of *MMP1* −1607 1G/2G, *MMP2* −1306 C/T, *MMP2* −1575 G/A, and *MMP9* Q279R were found between patients with POAG and control subjects and patients with XFG and control subjects, respectively (p>0.05). The presence of POAG or XFG was not predicted by any of the investigated polymorphisms.

**Conclusions:**

Our data suggest that the *MMP1* −1607 1G/2G, *MMP2* −1306 C/T, *MMP2* −1575 G/A, and *MMP9* Q279R polymorphisms themselves are unlikely major risk factors among Caucasian patients with either POAG or XFG.

## Introduction

Primary open angle glaucoma (POAG) and open angle glaucoma secondary to exfoliation syndrome (XFG) are among the main causes of irreversible blindness worldwide [1]. They are characterized by progressive loss of retinal ganglion cells (RGCs) and their respective axons leading to the pathognomonic cupping of the optic nerve head [2]. Although an elevated intraocular pressure, as a result of alterations in the trabecular meshwork (TM), remains the most important and presently only therapeutically modifiable risk factor, other risk factors including vascular, excitotoxic, neurotoxic, or genetic factors have been reported [3-5]. The impact of genetic factors has been studied with great effort leading to the identification of several mutations and gene polymorphisms as risk factors. Mutations in the myocilin gene (*MYOC*) in the case of POAG and as for XFG polymorphisms in the lysyl oxidase like protein 1 (*LOXL1*) gene are the most prominent among them [6-16]. However, mutations in *MYOC* are not found in most of patients with POAG and many individuals with polymorphisms in *LOXL1* do not develop exfoliation syndrome or XFG [17,18]. Therefore, other as yet unknown genetic factors might also play a pathogenetic role in these entities.

Matrix metalloproteinases (MMPs) comprise a family of at least 25 secreted zinc proteinases, which are of eminent importance not only for the extracellular matrix (ECM) turnover, but also for interactions between cells and their sorrounding [19]. They have been linked to many physiologic as well pathologic events (i.e., embryogenesis, wound healing and tissue remodeling, inflammation, angiogenesis, carcinogenesis). Not surprisingly, MMPs have also been demonstrated to exert pathogenetic effects in POAG and XFG. In a human outflow model injection of MMP2, MMP3, and MMP9 led to a significantly increased outflow facility [20], furthermore in an in vitro study using porcine TM cells mechanical stress increased MMP2 and MMP14 expression [21]. Interestingly, latanoprost has been suggested to exert its effects on the remodeling of the ECM in the TM via MMP2 and MMP3 [22]. In patients with POAG and XFG significantly altered levels of MMPs in the aqueous humor have been described [23,24]. In the optic nerve heads of both human glaucomatous eyes and monkey eyes with experimental glaucoma increased expression of MMP1 has been reported [25,26]. Furthermore, after optic nerve ligation MMP9 expression in the mouse retina is increased leading to apoptotic ganglion cell loss [27].

Generally, activity of MMPs is regulated at the level of expression, posttranslational activation, and inhibition [19]. With regard to expression of MMPs common functional polymorphisms have been identified. In the promotor region of *MMP1* an additional guanine at position −1607 (*MMP1* −1607 1G/2G; rs1799750) leads to a significantly higher transcription rate of the gene [28], while in the promotor region of *MMP2* a C to T transition at position −1306 (*MMP2* −1306 C/T; rs243865) as well as a G to A transition at position −1575 (*MMP2* −1575 G/A; rs243866) are associated with reduced transription activity [29,30]. A nonsynonymous A to G transition in exon 6 of *MMP9* leading to a substitution of arginine by glutamine at position 279 (*MMP9* Q279R; rs17576) has been shown to affect the substrate binding capacity [31-33]. Recently, an association of *MMP9* Q279R with acute primary angle closure glaucoma in Taiwanese patients has been found. Two subsequent studies conducted in Singapore and southern China were unable to replicate this finding [34-36]. As for the *MMP1* −1607 1G/2G polymorphism, a Greek study showed a trend for association with exfoliation syndrome [37].

Our study was set to investigate a hypothesized association of the aforementioned polymorphisms with POAG and XFG in a Central European population of Caucasian descent.

## Methods

In the present case-control study we investigated a total of 772 unrelated Caucasian subjects comprising 322 patients with POAG, 202 patients with XFG, and 248 control subjects. All participants were seen at the Department of Ophthalmology, Medical University Graz and gave written informed consent before enrolment. The study was conducted in accordance with the standards of the local Ethics Committee and the National Gene Technology Act.

All patients underwent slit lamp biomicroscopy, testing for best corrected visual acuity, Goldmann applanation tonometry, gonioscopy, and standard automated perimetry (Interzeag Octopus 101, program G2) or – in cases of profoundly decreased visual acuity – Goldmann perimetry. In all patients photographs of the optic disc were taken.

POAG was defined by an intraocular pressure before initiation of a pressure-lowering therapy of at least 21 mmHg, an open anterior chamber angle, optic disk changes characteristic for glaucoma (notching, thinning of the neuroretinal rim, increased cup/disc ratio in relation to the optic disc size), visual field defects characteristic for glaucoma (inferior or superior arcuate scotoma, nasal step, paracentral scotoma), and absence of conditions leading to secondary glaucoma. XFG was defined by an intraocular pressure before initiation of a pressure-lowering therapy of at least 21 mmHg, an open anterior chamber angle, optic disk changes, and visual field defects characteristic for glaucoma and presence of typical exfoliation material on the anterior lens capsule.

The control group consisted of 248 unrelated patients with no morphological or functional damage indicative for primary or secondary open angle or angle closure glaucoma. Control subjects were admitted to our department for cataract surgery. All participants were Caucasians from the same geographic area (Southern Austria). Individuals with significant co-morbidity for eye diseases (except cataract or mild diabetic retinopathy) were excluded from the study.

### Genotype determination

Venous blood was collected in 3 ml EDTA tubes. DNA was extracted form peripheral lymphocytes using the nucleic isolation kit: QIAamp DNA Mini and Blood Kit (Qiagen, Venlo, Netherlands) following the manufactures protocol and stored at −20 °C. Genotype determination was performed using high-resolution melting curve analysis on the LightCycler® 480 PCR system (Roche Diagnostics AG, Risch, Switzerland). The samples were amplified in duplicate 20 µl reactions using the Light Cycler 480 High Resolution Melting Master kit (Roche Diagnostics, Wien, Austria) and analyzed on a LC480 instrument I (Roche Diagnostics GmbH, Mannheim, Germany). The final reaction mix contained 1× Master Mix, 3 mM MgCl_2_, 4 µM forward and reverse primer (Table 1) and 50 ng of genomic DNA. For PCR the following cycling conditions were chosen: one cycle of 95 °C for 10 min followed by 45 cycles of 95 °C for 10 s, 58 °C-60 °C for 15 s depending on the SNP investigated (58 °C - MMP-9-rs17576, 60 °C - MMP-1-rs1799750, MMP-2-rs243865, MMP-2-rs243866) and 72 °C for 20 s. The amplicons were then denatured at 95 °C for 1 min, cooled down to 40 °C for 1 min and then melted from 65 °C to 95 °C with 25 signal acquisitions per degree. To detect sequence variations the Gene Scanning Software v1.5 (Roche Diagnostics GmbH) was used. Using the Auto Group mode samples were automatically grouped because of their melting curves.

**Table 1:** Nucleotide sequences, melting temperature, and product sizes.

**Name of primer**	**Nucleotide sequence**	**Melting temperature**	**Product size**
MMP1-1607 1G/2G left	5'-tgccacttagatgaggaaatt g-3'	59.22 °C	127 bp
MMP1-1607 1G/2G right	5'-cctgttttctttctgcgtca-3'	59.05 °C	
MMP2-1306 C/T left	5'-tttttcatctctgggccatt-3'	59.50 °C	101 bp
MMP2-1306 C/T right	5'-gacttctgagctgagacctga-3'	57.30 °C	
MMP2-1575 G/A left	5'-gtctgaagcccactgagacc-3'	59.84 °C	121 bp
MMP2-1575 G/A right	5'-aggtcaggggctgaaagaat-3'	60.07 °C	
MMP9 R279Q A/G left	5'-caggacacacttgggggtta-3'	60.80 °C	136 bp
MMP9 R279Q A/G right	5'-gccttggaagatgaatggaa-3'	60.01 °C	

### Statistical analysis

Descriptive statistics were used to calculate frequencies and percentages of discrete variables. Continuous data are given as mean ±standard deviation (SD). Means were compared using Mann–Whitney test. Proportions of groups were compared by χ^2^ test. Odds ratio (OR) and 95% confidence interval (95%CI) were calculated by logistic regression. Assuming a codominant effect, genotypes were coded as 0 (no variant allele, wildtype), 1 (one variant allele, heterozygous genotype), or 2 (two variant alleles). Hardy–Weinberg equilibrium has been calculated using HW Diagnostics-Version 1.beta (Fox Chase Cancer Center, Philadelphia, PA). Statistical analysis was done using the SPSS statistical package (SPSS, version 17.0, Chicago, IL). Power calculation was done using PS Power and Sample Size Calculation software version 2.1.30 [38].

## Results

Our study included 322 patients with POAG (188 female and 134 male), 202 patients with XFG (112 female and 90 male), and 248 control subjects (126 female and 122 male). Clinical characteristics of patients and control subjects are shown in Table 2 and Table 3. Patients with POAG had a mean deviation of 12.3±6.4 dB, a mean loss of variance of 40.0±27.3 square decibel, while patients with XFG had a mean deviation of 12.7±6.8 dB, a mean loss of variance of 36.5±22.1 square decibel.

**Table 2:** Clinical characteristics of patients with POAG and controls.

**Clinical characteristic**	**Patients with POAG (n=322)**	**Control subjects (n=248)**	**Significance p-value**
Mean age (±SD)	74.1±10.6	74.4±7.1	0.19
Range (years)	37.1–92.9	57.2–90.7	
Arterial hypertension*	194 (60.2)	146 (58.9)	0.80
Diabetes mellitus*	36 (11.2)	32 (12.9)	0.60

*Numbers are given as n (%).

**Table 3:** Clinical characteristics of patients with XFG and controls.

**Clinical characteristic**	**Patients with XFG (n=202)**	**Control subjects (n=248)**	**Significance p-value**
Mean age (±SD)	75.1±7.1	74.4±7.1	0.15
Range (years)	50.3–87.0	57.2–90.7	
Arterial hypertension*	116 (57.4)	146 (58.9)	0.77
Diabetes mellitus*	20 (9.9)	32 (12.9)	0.38

*Numbers are given as n (%).

Genotype distributions or allelic frequencies of *MMP1* −1607 1G/2G, *MMP2* −1306 C/T, *MMP2* −1575 G/A, and *MMP9* Q279R did not significantly differ between patients with POAG and control subjects (Table 4).

**Table 4:** Genotype and allele frequencies in patients with primary open angle glaucoma (POAG).

**Single nucleotide polymorphism**	**Patients with POAG (n=322)**	**Control subjects (n=248)**	**p-value**
*MMP1* −1607 1G/1G	89 (27.6%)	60 (24.2%)	0.35
1G/2G	165 (51.2%)	131 (52.8%)	
2G/2G	68 (21.1%)	57 (23.0%)	
2G frequency	0.467	0.494	0.37
*MMP2* −1306 C/C	187 (58.1%)	138 (55.6%)	0.56
C/T	111 (34.5%)	88 (35.5%)	
T/T	24 (7.5%)	22 (8.9%)	
T allele frequency	0.247	0.266	0.46
*MMP2* −1575 G/G	185 (57.5%)	142 (57.3%)	0.96
G/A	117 (36.3%)	86 (34.7%)	
A/A	20 (6.2%)	20 (8.1%)	
A allele frequency	0.244	0.254	0.69
*MMP9*rs17576 A/A	139 (43.2%)	102 (41.1%)	0.63
A/G	141 (43.8%)	120 (48.4%)	
G/G	42 (13.0%)	26 (10.5%)	
G allele frequency	0.349	0.347	0.93

Numbers for genotypes are n (%).

Similarly, no significant differences in either genotype distributions or allelic frequencies of *MMP1* −1607 1G/2G, *MMP2* −1306 C/T, *MMP2* −1575 G/A, and *MMP9* Q279R were found between patients with XFG and control subjects (Table 5). In a logistic regression analysis, presence of either POAG or XFG was not predicted by any of the investigated polymorphisms (Table 6).

**Table 5:** Genotype and allele frequencies in patients with open angle glaucoma secondary to exfoliation syndrome (XFG).

**Single nucleotide polymorphism**	**Patients with XFG (n=202)**	**Control subjects (n=248)**	**p-value**
*MMP1* –1607 1G/1G	51 (25.2%)	60 (24.2%)	0.80
1G/2G	96 (47.5%)	131 (52.8%)	
2G/2G	55 (27.2%)	57 (23.0%)	
2G frequency	0.510	0.494	0.63
*MMP2* −1306 C/C	107 (53.0%)	138 (55.6%)	0.57
C/T	80 (39.6%)	88 (35.5%)	
T/T	15 (7.4%)	22 (8.9%)	
T allele frequency	0.272	0.266	0.84
*MMP2* −1575 G/G	109 (54.0%)	142 (57.3%)	0.48
G/A	82 40.6%)	86 (34.7%)	
A/A	11 (5.4%)	20 (8.1%)	
A allele frequency	0.257	0.254	0.91
*MMP9*rs17576 A/A	88 (43.6%)	102 (41.1%)	0.60
A/G	83 (41.1%)	120 (48.4%)	
G/G	31 (15.3%)	26 (10.5%)	
G allele frequency	0.359	0.347	0.71

Numbers for genotypes are n (%).

**Table 6:** Logistic regression analysis of primary open angle glaucoma (POAG) and open angle glaucoma secondary to exfoliation syndrome (XFG) risk.

**Single nucleotide polymorphism**	**Odds ratio**	**95% Confidence Interval**	**p-value**
**POAG**			
*MMP1* −1607 2G	0.90	0.71–1.14	0.40
*MMP2* −1306 T	0.90	0.69–1.18	0.49
*MMP2* −1575 A	0.95	0.72–1.24	0.73
*MMP9*rs17576 G	1.01	0.79–1.29	0.95
**XFG**			
*MMP1* −1607 2G	1.07	0.82–1.39	0.64
*MMP2* −1306 T	1.03	0.77–1.39	0.88
*MMP2* −1575 A	1.02	0.75–1.38	0.94
*MMP9*rs17576 G	1.06	0.80–1.39	0.73

The observed genotype distributions did not deviate from those predicted by the Hardy–Weinberg equilibrium.

## Discussion

Elevated intraocular pressure generated by increased outflow resistance leading to apoptotic death of retinal ganglion cells is the main risk factor in the pathogenesis of POAG and XFG. Increased plaque-like material within the TM as well as loss of TM cells have been linked to the increased outflow resistance [39,40], whereas glaucomatous RGC death occurs by apoptosis, a subacute process provoked by cellular degradation rather than disruption [41,42]. The exact biochemical pathways, however, leading to accumulation of ECM in the TM as well as apoptotic death of retinal ganglion cells are not understood. In the last years diverse studies provided evidence that MMPs may be involved in both pathomechanisms. For example, in an ex vivo model MMP2, MMP3, and MMP9 led to an enhanced outflow facility, while MMP1 concentrations were significantly elevated in glaucomatous optic nerve heads [20,25]. Furthermore, in a rodent glaucoma model optic nerve ligation led to significantly increased MMP9 concentrations in the retina [27].

In the present study genotypes of the functional *MMP1* −1607 1G/2G, *MMP2* −1306 C/T, *MMP2* −1575 G/A, and *MMP9* Q279R polymorphisms were determined in 322 patients with POAG, 202 patients with XFG and 248 control subjects. No significant differences were found in the genotype distributions or allelic frequencies of the investigated polymorphisms between patients and control subjects. As the study has a statistical power of 0.80 to detect odds ratios between 1.61 and 1.68 for the allelic variants of the *MMP1* −1607 1G/2G, *MMP2* −1306 C/T, *MMP2* −1575 G/A, and *MMP9* Q279R polymorphisms in patients with POAG and odds ratios between 1.71 and 1.77 in patients with XFG, respectively, our data suggest that the investigated polymorphisms are unlikely major genetic risk factors for POAG and XFG in Caucasian patients.

Remarkably, investigating the association between exfoliation syndrome and XFG and the *MMP1* −1607 1G/2G polymorphism, Tsironi and coworkers [37] reported an allele contrast of borderline significance for exfoliation syndrome (OR=1.47; 95%CI: 1.03–2.10; p=0.04), but not for XFG. Ninety patients with exfoliation syndrome and 92 patients with XFG were included in their study. Furthermore, Wang and coworkers [34-36] reported a significant association between *MMP9* Q279R and acute primary angle closure glaucoma in Taiwanese patients (OR=2.59; 95%CI: 1.71–3.90; p=<0.001), albeit this result could not be replicated by subsequent studies from Singapore and southern China.

According to their far-reaching impact on the protein degradation of the ECM as well as on the interaction of cells with their surrounding, regulation of MMPs is under tight control and is achieved on different levels. Besides epigenetic regulation via histone modification and chromatin-remodeling complexes, evidence emerged that post-translational mechanisms leading to altered stability of mRNA are also involved [43]. For example, transforming growth factor-beta (TGF-β) extends the half-life of mRNAs of *MMP2* and *MMP9* significantly, thereby increasing MMP2 and MMP9 concentrations [44]. Furthermore, MMPs are secreted as inactive proenzymes and can be activated by other proteinases including active MMPs [19]. Ultimately, inhibition of MMPs is accomplished by specific (i.e., tissue inhibitor of metalloproteinases) and unspecific (e.g., α2-macroglobulin) inhibitors [45,46]. Thus, our finding that the *MMP1* −1607 1G/2G, *MMP2* −1306 C/T, *MMP2* −1575 G/A, and *MMP9* Q279R polymorphisms are not associated with an increased risk for POAG or XFG does not exclude a substantial role of *MMP1*, *MMP2*, and *MMP9* in the pathogenesis of POAG or XFG.

In conclusion, based on the results of the present study, none of the investigated functional polymorphisms in *MMP1*, *MMP2*, and *MMP9* themselves are major risk factors among Caucasian patients with POAG or XFG.
